# Sensitization of *Staphylococcus aureus* to Methicillin and Other Antibiotics *In Vitro* and *In Vivo* in the Presence of HAMLET

**DOI:** 10.1371/journal.pone.0063158

**Published:** 2013-05-01

**Authors:** Laura R. Marks, Emily A. Clementi, Anders P. Hakansson

**Affiliations:** 1 Department of Microbiology and Immunology, University at Buffalo, State University of New York, Buffalo, New York, United States of America; 2 The Witebsky Center for Microbial Pathogenesis and Immunology, University at Buffalo, State University of New York, Buffalo, New York, United States of America; 3 New York State Center of Excellence in Bioinformatics and Life Sciences. University at Buffalo, State University of New York, Buffalo, New York, United States of America; Rockefeller University, United States of America

## Abstract

HAMLET (human alpha-lactalbumin made lethal to tumor cells) is a protein-lipid complex from human milk with both tumoricidal and bactericidal activities. HAMLET exerts a rather specific bactericidal activity against some respiratory pathogens, with highest activity against *Streptococcus pneumoniae*, but lacks activity against most other bacterial pathogens, including Staphylococci. Still, ion transport associated with death in *S. pneumoniae* is also detected to a lower degree in insensitive organisms. In this study we demonstrate that HAMLET acts as an antimicrobial adjuvant that can increase the activity of a broad spectrum of antibiotics (methicillin, vancomycin, gentamicin and erythromycin) against multi-drug resistant S*taphylococcus aureus,* to a degree where they become sensitive to those same antibiotics, both in antimicrobial assays against planktonic and biofilm bacteria and in an *in vivo* model of nasopharyngeal colonization. We show that HAMLET exerts these effects specifically by dissipating the proton gradient and inducing a sodium-dependent calcium influx that partially depolarizes the plasma membrane, the same mechanism induced during pneumococcal death. These effects results in an increased cell associated binding and/or uptake of penicillin, gentamicin and vancomycin, especially in resistant stains. Finally, HAMLET inhibits the increased resistance of methicillin seen under antibiotic pressure and the bacteria do not become resistant to the adjuvant, which is a major advantageous feature of the molecule. These results highlight HAMLET as a novel antimicrobial adjuvant with the potential to increase the clinical usefulness of antibiotics against drug resistant strains of *S. aureus*.

## Introduction

Methicillin-resistant *Staphylococcus aureus* (MRSA) is one of the principal multi-drug resistant bacterial pathogens causing serious community and hospital-acquired infections [1]–[3], such as skin and soft tissue infections, bone, joint and implant infections, ventilator-associated pneumonia, and sepsis [4]. It is estimated that multi-drug resistant *Staphylococcus aureus* infections leads to 19,000 deaths per year in the United States, with an associated 3-4 billion US dollars in annual health care costs [5], [6]. Despite this high mortality rate, there are relatively few new antibacterial agents in the pharmaceutical pipeline [7]. Instead, the majority of antibiotics developed in the last decade are molecules re-engineered from existing antibiotic classes for which underlying resistance mechanisms are already present [8]. Therefore effective new therapeutic options for treatment of infections caused by multi-drug resistant *S. aureus* are urgently needed.

One attractive strategy would be the reintroduction of current and previously used antibiotics to which MRSA strains are resistant, when used in combination with other sensitizing agents. These ‘antimicrobial adjuvants’ may not have significant antibiotic activity alone, however would improve the biologic activity of antibiotics when used in combination [9]–[12]. Introduction of such antimicrobial adjuvants would provide a new dimension of safe and widely available treatment possibilities.

Human milk contains numerous antimicrobial and immunomodulatory factors [13]–[15]. We have identified and characterized one of them, HAMLET (Human α-lactalbumin made lethal to tumor cells), a complex of human milk alpha-lactalbumin and the fatty acids oleic (C18:1) and linoleic acid (C18:2) [16], [17] that constitutes around 60% of fatty acids in human milk [18], [19]. HAMLET was originally isolated from human milk casein and had a broad anti-tumor activity without affecting healthy cells [20]. Later it was shown that HAMLET's anti-tumor activity was due to internalization of the HAMLET-complex only in tumor cells, where it co-localized with mitochondria, resulting in a calcium-dependent depolarization of the inner membrane and induction of apoptosis [21], 22.

Based on the evolutionary association between mitochondria and bacteria we tested HAMLET's effects on bacteria and showed that HAMLET had bactericidal activity primarily against some respiratory tract pathogens, such as *Streptococcus pneumoniae* [23]. HAMLET kills pneumococci by inducing a sodium-dependent influx of calcium ions [24] that leads to depolarization and execution of cell death with morphological and biochemical similarities to eukaryotic apoptosis [25]. Recently, we showed that HAMLET at sublethal concentrations could potentiate the effect of antibiotics against *S. pneumoniae* using the same mechanism and that this potentiation also worked against other respiratory tract pathogens [26]. HAMLET has no bactericidal activity against Staphylococci, but still induce some level of depolarization [24], indicating transport of ions over the bacterial membrane.

In this paper, we describe HAMLET's antimicrobial adjuvant activity against *Staphylococcus aureus* and the specific role of HAMLET's membrane effects in this process. We demonstrate that HAMLET, by specifically dissipating the proton motive force and inducing sodium-dependent calcium transport, potentiates the activity of a broad spectrum of antibiotics, including methicillin, erythromycin, gentamicin and vancomycin on multi-drug resistant *S. aureus in vitro* and *in vivo*. We also show that HAMLET inhibits the development of clones with increased methicillin resistance when a MRSA population is exposed to increasing concentrations of the antibiotic and that the bacteria cannot overcome HAMLET's inhibitory effect. These results highlight the potential of HAMLET as a novel starting point for discovering new antimicrobial adjuvants to combat staphylococcal infections and increase the usefulness of formerly useful antibiotics, such as methicillin and penicillins.

## Results

### Sensitizing activity of HAMLET on antibiotics in vitro

A standard checkerboard broth microdilution assay was used to test whether HAMLET interfered with the susceptibility of bacteria to antibiotics that target cell wall synthesis (methicillin and vancomycin) as well antibiotics that target protein synthesis (erythromycin and gentamicin). HAMLET alone had no activity against any of the *S. aureus* strains tested even at concentrations exceeding 5,000 µg/ml.

**Figure 1:**
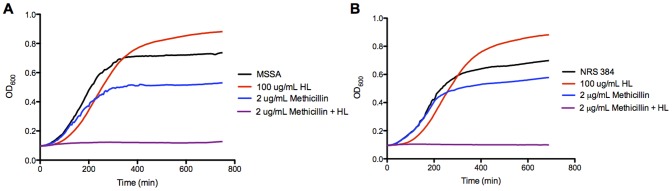
HAMLET lowers the methicillin MIC. *S. aureus* strains 11090306 (MSSA) (left) and NRS 384 (MRSA) (right) were grown in broth for 16 hours in the presence of 2 µg/ml methicillin (5 µM) with and without the addition of 100 µg/mL (6 µM) HAMLET. The figure shows representative growth curves for the lowest concentration of antibiotic and HAMLET that inhibited bacterial growth by combination treatment without either agent alone affecting growth.

However, in the presence of HAMLET-concentrations as low as 100 μg/mL (6 μM), all *S. aureus* strains tested showed 2 to >16 fold reductions in the minimal concentration that inhibited growth (MIC) of methicillin (**Figure 1** **,** **Table 1**), vancomycin, erythromycin and gentamicin (**Table 1**) and 2 to >32 fold reductions in the minimal bactericidal concentration (MBC) of these same antibiotics (**Table 1**). Some of the MIC and MBC-reductions may well be larger as our assay did not go beyond 128 and 256 µg/ml, respectively, at which concentration several strains still grew normally. The addition of 6 µM HAMLET in the assays decreased the MIC for methicillin more in resistant strains compared with the methicillin-sensitive strains of *S. aureus* (MSSA). Furthermore, for both MRSA and MSSA strains, the fold decrease in the MBC concentrations in the presence of HAMLET was generally more pronounced than the decrease in the MIC concentrations (**Table 1**). This was also observed when HAMLET was added in combination with other antibiotics, with the exception of gentamicin that showed a more pronounced reduction in MIC in the presence of HAMLET with less of an effect on the MBC (**Table 1**).

**Table 1:** MIC and MBC values for *S. aureus* strains exposed to various antibiotics in the absence and presence of HAMLET.

Bacterial Strain	Antibiotics, MIC (µg/ml)		MIC Fold reduction	Antibiotics, MBC (µg/ml)		MBC Fold reduction	[HL]** to sensitize, µM
	Meth	Meth + HL *		Meth	Meth + HL *		
**NRS1**	>128	16	>8	>256	64	>4	48
**NRS70**	32	1	32	128	4	32	3
**NRS71**	>128	16	>8	>256	64	>4	54
**NRS100**	>128	8	>16	>256	64	>4	18
**NRS123**	32	4	8	>256	16	>16	12
**NRS384**	16	2	8	>256	8	>32	6
**10307570**	16	2	8	>256	16	>16	6
**11090306**	2	1	2	64	4	16	0
	Erm	Erm + HL *		Erm	Erm + HL *		
**NRS384**	16	4	4	>256	16	>16	6
**NRS123**	0.5	0.25	2	16	4	4	0
	Gent	Gent + HL *		Gent	Gent + HL *		
**NRS384**	1	0.00625	160	16	4	4	0
**NRS1**	>128	8	>16	>256	32	>8	48
	Vanc	Vanc + HL *		Vanc	Vanc + HL *		
**NRS384**	1	0.5	2	16	8	2	0
**NRS1**	8	4	2	32	8	4	18

* HL  =  HAMLET. Concentration used was 100 µg/ml (6 µM).

** The last column describes the concentration of HAMLET required to make each strain sensitive to the respective antibiotic.

Although 6 µM of HAMLET could make three of the strains sensitive to methicillin and gentamicin (**Table 1**) this concentration was insufficient to make the remaining strains sensitive. However, using increased concentrations of HAMLET (requiring at most 54 µM), all antibiotic-resistant strains could be converted to methicillin, gentamicin, erythromycin and vancomycin sensitivity (**Table 1**, Last column).

Similar to the MIC and MBC assays, treatment of bacteria grown to mid-log phase (approximately 10^8^ CFU/ml) with antibiotics alone or in combination with HAMLET resulted in the same phenotype with immediate bacteriostatic effects followed by bactericidal activity after 6 hours of incubation at lower concentrations of antibiotics when HAMLET was present (not shown). Combined these results suggest that HAMLET potentiates the anti-staphylococcal effects of various classes of antibiotics with a significantly better potentiation seen in antibiotic-resistant strains (**Table 1**).

### HAMLET potentiates methicillin and vancomycin killing of in vitro biofilms

**Figure 2:**
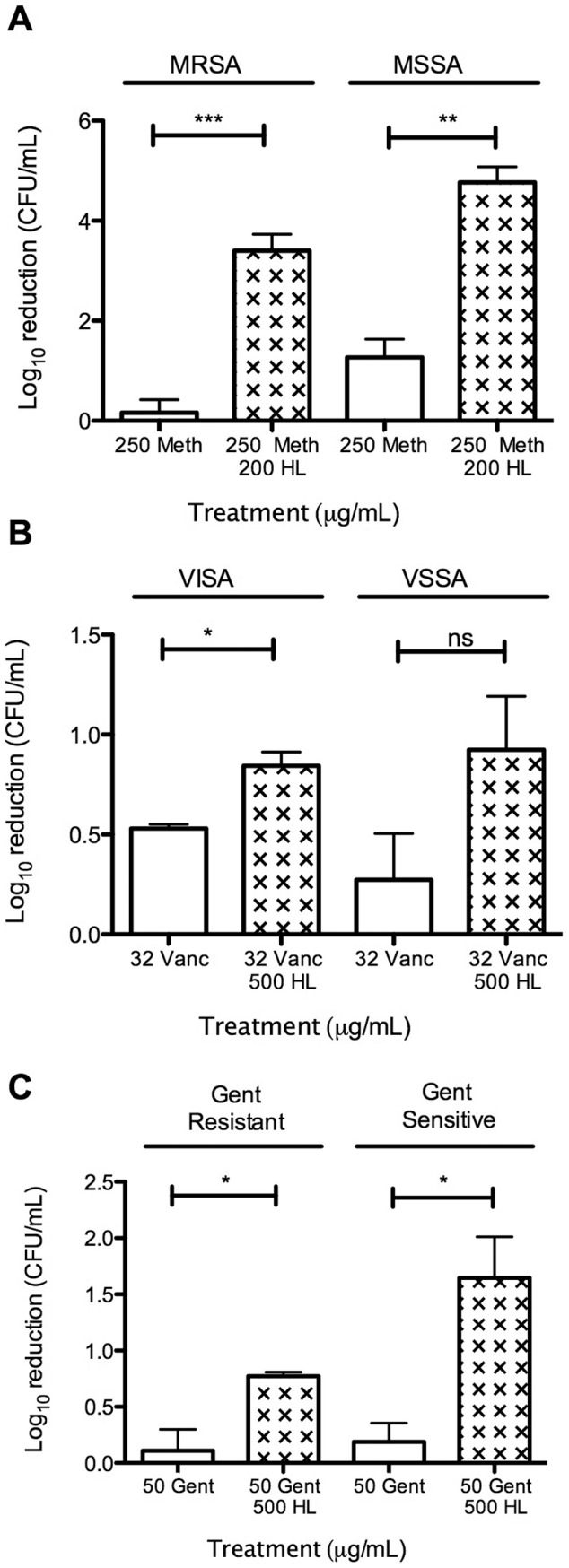
The effect of HAMLET/antibiotic combination treatment on *in vitro* biofilm viability. (A) The activity of methicillin (250 μg/mL or 660 µM), HAMLET (200 μg/mL or 12 µM), or the combination of both agents were tested on *in vitro* biofilms of the methicillin-resistant strain NRS 70 (MRSA) or the methicillin-sensitive strain 11090306 (MSSA) by determining the bacterial death (in log_10_) after culturing dilutions overnight on blood agar. (B) The activity of vancomycin (32 μg/mL or 21 µM), HAMLET (500 μg/mL or 30 µM), or the combination of both agents on *in vitro* biofilms of the vancomycin-resistant strain NRS 1 (VISA) and the vancomycin-sensitive strain NRS384 (VSSA) were tested in a similar fashion as was the activity of (C) 50 µg/ml (105 µM) gentamicin alone or in combination with 500 µg/ml (30 µM) HAMLET for the gentamicin-resistant strain. The results are based on three independent experiments with duplicate samples. Statistics was performed using the paired Student t-test. Significance was indicated as follows: ns  =  not significant, *  =  *P*<0.05, **  =  *P*<0.01.

To address the role of HAMLET's effect on antibiotic function under more physiological conditions, we first investigated the potentiating effects of HAMLET on antibiotic activity against *S. aureus* biofilms. *S. aureus* biofilms were formed with the MRSA strain NRS 70 and the MSSA strain 11090306 overnight on polystyrene plates. As bacterial biofilms, including staphylococcal biofilms, are inherently resistant to most antimicrobials [27], [28], higher concentrations of each agent were required to see bactericidal effects. Using either 200 μg/mL (12 μM) of HAMLET or 250 μg/mL (660 µM) of methicillin alone failed to induce bactericidal activity over a 24-hour period (**Figure 2A**), whereas a bactericidal effect (>3 log_10_ reduction in the number of viable bacteria) was observed against both the MRSA and the MSSA biofilms when the agents were combined (**Figure 2A**).

HAMLET was less active when used in combination with vancomycin against biofilms formed by the vancomycin-insensitive *S. aureus* (VISA) strain NRS 1 or the vancomycin-sensitive *S. aureus* (VSSA) strain NRS 384. Biofilms formed by these strains showed almost complete insensitivity to treatment with 32 µg/mL (21 µM) vancomycin alone, similar to published data [27], however the combination of HAMLET and vancomycin demonstrated an increased death of staphylococcal biofilm bacteria over 24 hours that was significant for NRS 1 (*P*<0.05), but not for NRS 384 (**Figure 2B**).

Finally, we tested the effect of gentamicin in combination with HAMLET against the gentamicin resistant strains NRS 1 and the gentamicin-sensitive strains NRS 384. Neither HAMLET (500 µg/ml; 30 µM) nor gentamicin (50 µg/ml; 105 µM) had any effect on the viability of either strain alone but in combination they produced significantly increased loss of bacterial viability (*P*<0.05 for both strains; **Figure 2C**).

### Reduction of nasal MRSA colonization by HAMLET and methicillin in vivo

**Figure 3:**
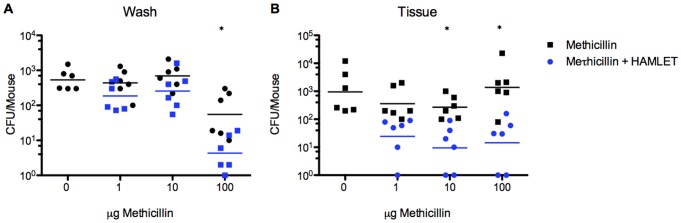
HAMLET/Methicillin combination treatment reduces Staphylococcal nasopharyngeal colonization. Mice were colonized with *S. aureus* NRS 70 for 24 hours, treated intranasally with various doses of gentamicin in the presence (blue) or absence (black) of HAMLET (100 µg) for 12 hours, and the bacterial burden associated with the nasal wash (A) and the nasopharyngeal tissue (B) was determined. The graph shows colonization data for individual mice, with the mean recovered bacteria depicted with a line. The results are based on experiments using groups of 6 mice. Statistics was performed using the unpaired Student t-test. Significance was indicated as follows: *  =  *P*<0.05.

To determine the efficacy of HAMLET/antibiotic combination treatment against MRSA colonization *in vivo*, mice were colonized with MRSA NRS 70 for 24 h and treated with one dose of either buffer (PBS) or methicillin in the presence or absence of HAMLET (**Figure 3**). Even administration of 100 µg of methicillin failed to significantly reduce the bacterial burden associated with the nasal mucosa compared with the buffer-alone treated control group. In contrast, administration of similar a amount of methicillin in combination with 100 μg HAMLET caused a significant decrease in MRSA colonization in the nasopharyngeal tissue (*P*<0.05). This effect was also detected when a lower dose of methicillin (10 µg) was tested in the presence of 100 µg of HAMLET (*P*<0.05).

### HAMLET adjuvant activity requires sodium dependent calcium influx

HAMLET's bactericidal activity on pneumococci is associated with a sodium-dependent calcium influx that that is also induced in a range of bacterial species, including Staphylococci [24], [26], where this influx does not result in death activation. We therefore hypothesized that this ion transport mechanism could play a role in HAMLET-induced sensitization in *S. aureus.*

**Figure 4:**
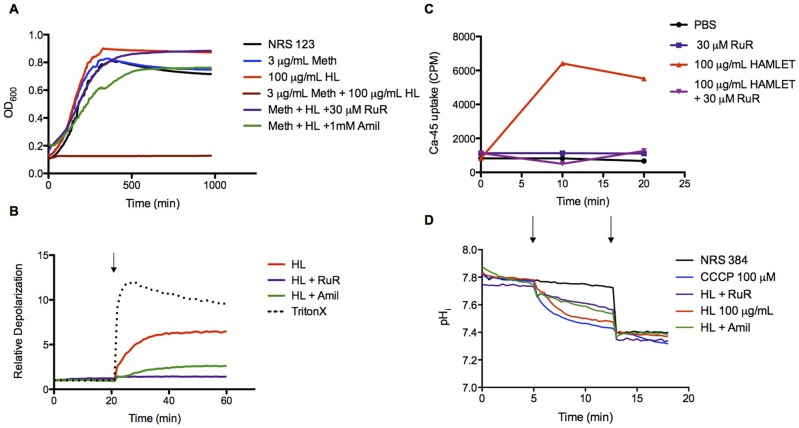
Effect of HAMLET on membrane potential. (A) Representative growth curves for *S. aureus* strain NRS 123 (MRSA) grown in broth for 16 hours (960 min) in the presence of methicillin with and without the addition of HAMLET and the inhibitors Ruthenium Red (RuR) or Amiloride (Amil). (B) Mid-log phase grown NRS 123 Staphylococci re-suspended in PBS alone or PBS plus Amiloride or Ruthenium Red, were incubated with the fluorescent indicator dye DiBAC_4_(3) and membrane depolarization was detected by measuring fluorescence over time. HAMLET was added at twenty minutes (arrow). The detergent Triton X-100 (0.1%) was included as a positive control. The results presented are from one representative experiment. (C) Mid-log phase grown NRS 384 Staphylococci were incubated with the radioisotope ^45^Ca^2+^ (2.5 µCi/mL) in PBS or PBS + Ruthenium Red (30 µM). After recording baseline readings, PBS (untreated), or HAMLET was added (Time  = 0 min) to the bacteria and radioactivity was measured over time. Results from a representative experiment are shown. (D) NRS 384 Staphylococci were loaded with the pH sensitive dye BCECF-AM, and were washed and resuspended in PBS + 25 mM glucose. After recording baseline readings, at the first arrow, PBS (untreated), the protonophore CCCP (100 µM), HAMLET (100 µg/mL or 6 µM), HAMLET + RuR (30 µM), or HAMLET + Amiloride (1 mM) were added to the bacteria and fluorescence was measured over time. At the second arrow 20 µM each of nigericin and valinomycin was added to completely dissipate the transmembrane proton gradient.

We first performed an MIC assay in the presence of the calcium transport inhibitor Ruthenium Red (RuR) and the sodium transport inhibitor Amiloride that reduces the calcium influx and loss of membrane potential in *S. pneumoniae* in response to HAMLET [24], [26], We found that the addition of either inhibitor completely abolished HAMLET's antibiotic potentiation effect on Staphylococci (**Figure 4A**), as well as the membrane depolarization associated with HAMLET-potentiation (**Figure 4B**). To confirm that the HAMLET-induced membrane depolarization was associated with Ca^2+^ transport we directly monitored uptake of Ca^2+^ using the radioisotope ^45^Ca^2+^. Intracellular Ca^2+^ rose immediately upon HAMLET addition, and was almost completely blocked in RuR treated cells (**Figure 4C**).

This effect was unrelated to any effects on membrane integrity, as HAMLET caused no membrane damage, indicated by a lack of propidium iodide staining of bacterial DNA inside the cells, or of leakage of ATP, DNA, or RNA into the culture supernatant (measured by a luciferase assay, or the absorbance of the supernatant at 260 nm, respectively, as described in the Materials section; data not shown). This was not surprising as HAMLET is unable to kill *S. aureus* even at concentrations 50-fold higher than those used in these assays. These results suggest that HAMLET-induced sensitization of *S. aureus* requires sodium-dependent calcium influx resulting in rapid depolarization of the transmembrane electrical potential of the staphylococcal membrane and is not associated with membrane disruption (**Figure 4C**).

### HAMLET induces dissipation of the proton motive force

The observed effect of HAMLET on membrane potential prompted us to examine other aspects of membrane function associated with antibiotic resistance. The proton motive force is composed of both the transmembrane electrical potential measured above, and the transmembrane chemical proton gradient both of which are important for drug efflux [29], [30]. To assess the effect of HAMLET on the transmembrane proton gradient we measured the intracellular pH of cells after exposure to HAMLET. A representative experiment is shown in **Figure 4D**. As a positive control the hydrogen-ionophore CCCP was added (1^st^ arrow – blue line). Addition of HAMLET (1^st^ arrow – red line) similarly resulted in rapid intracellular acidification and dissipation of the pH gradient. Preincubation of cells with Ruthenium red (purple line) or Amiloride (green line) did not fully inhibit HAMLET-induced dissipation of the pH gradient (1^st^ arrow), indicating that sodium-dependent calcium transport and dissipation of the proton gradient may be partially parallel or sequential. At the end of each experiment (2^nd^ arrow) the pH gradient was completely dissipated using a combination of nigericin and valinomycin to induce a pH_i_ of the external buffer (complete dissipation of the proton motive force). The results show that HAMLET effectively dissipated the proton gradient.

### HAMLET increases gentamicin, vancomycin, and beta lactam association with staphylococcal cells

**Figure 5:**
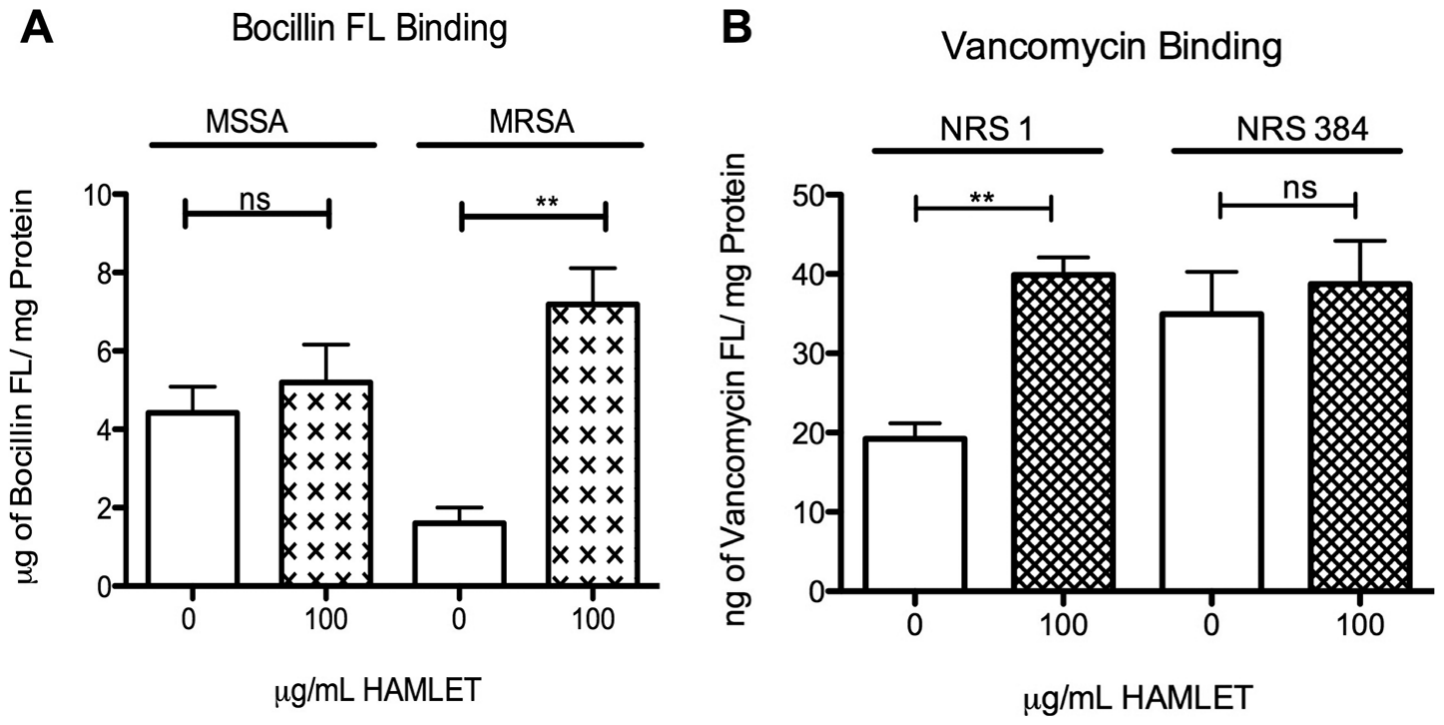
Impact of HAMLET on uptake and binding of Bocillin FL and vancomycin FL. (A) Staphylococci were incubated with Bocillin FL or (B) with vancomycin FL in the presence or absence of 100 µg/mL (6 µM) HAMLET. The results are based on three individual experiments with duplicate samples. Statistics was performed using the unpaired Student's t-test. Significance was indicated as follows: **  =  P<0.01, ***  =  P<0.001, ns  =  not significant.

As HAMLET dissipated the proton motive force, which is tightly associated with multidrug efflux pump function, we evaluated the effect of HAMLET-treatment on the binding/uptake of fluorescently labeled gentamicin, vancomycin and the beta-lactam Bocillin FL (a fluorogenic derivative of penicillin V). In keeping with the results of our MIC assays we found that the addition of 100 μg/mL HAMLET resulted in no significant increase in Bocillin FL association with the methicillin-sensitive strain, whereas HAMLET-addition to the methicillin-resistant strain NRS 384 caused a significantly increased Bocillin FL-association compared with the Bocillin FL alone treated culture (*P*<0.01; **Figure 5A**).

Similarly, the addition of HAMLET did not increase the cell-associated levels of vancomycin in the vancomycin-sensitive strain but significantly increased the association with the VISA strain NRS 1 (*P*<0.01; **Figure 5B**). The addition of HAMLET also increased the cell-associated level of gentamicin 2.6 fold in the NRS384 strain, although not to a significant degree (data not shown, *P* = 0.09). Combined these results suggest that HAMLET's effect on membrane function resulted in an increased association of antibiotics with the bacterial cells particularly for resistant strains.

### HAMLET suppresses methicillin-resistance development in *S. aureus*, and *S. aureus* does not develop resistance to HAMLET adjuvant activity

Antibiotic resistance is induced and enhanced with repeated exposure to increasing concentration of antibiotics [31], which may well have implications during clinical treatment. Thus, reducing resistance development upon repeated exposure would confer significant clinical benefits. To evaluate HAMLET's potential effect on resistance development, experiments were designed to test the impact of HAMLET on the resistance-development of MRSA to methicillin in liquid cultures.

**Figure 6:**
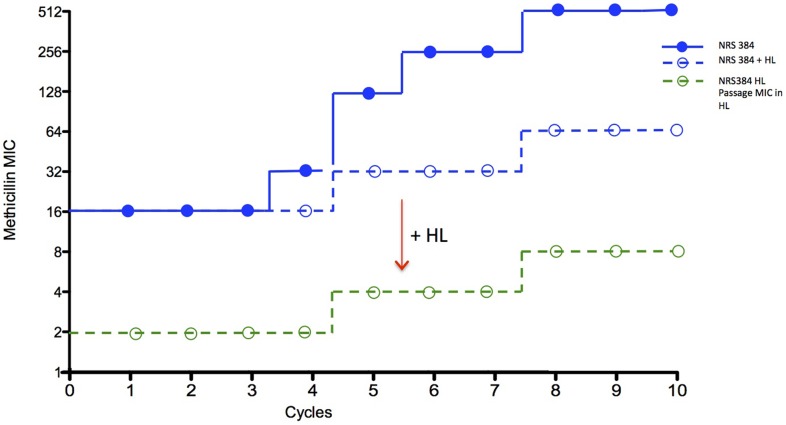
HAMLET and resistance development. Methicillin adaptation of the MRSA strain NRS 384 after exposure to stepwise increasing concentrations of methicillin alone (1–512 µg/mL or 2.5–1,350 µM) or methicillin in combination with 100 µg/mL (6 µM) HAMLET. The filled blue circles show the methicillin MICs after each cycle when no HAMLET was used. The addition of 100 µg/mL of HAMLET reduced methicillin-induced resistance (blue unfilled circles). The unfilled green circle represents the MIC of methicillin of the bacteria grown in presence of HAMLET, when HAMLET was also present during the MIC assay to potentiate the effect of the antibiotic. Reintroduction of HAMLET to these isolates again returned the methicillin MIC back to the levels denoted by the green unfilled circle.

For NRS 384 cultures grown in the absence of HAMLET, the MIC of methicillin increased from 16 µg/ml to 512 μg/mL after 8 cycles of sequential incubation with increasing concentrations of methicillin (**Figure 6** – blue line). Surprisingly, the addition of a low concentration of HAMLET (100 μg/mL; 6 µM) drastically reduced this increased methicillin-resistance of the strain. After 10 cycles this culture had a methicillin MIC of only 64 μg/mL (**Figure 6** **–** hatched blue line). When HAMLET was present during the MIC testing, the highest concentration of methicillin that the strain was able to grow in was 8 μg/ml, the same fold reduction (8-fold) as observed before exposing the strain to cycles of increasing antibiotic concentrations, demonstrating that no resistance to HAMLET's adjuvant activity occurred even after continuous incubation in 100 μg/mL HAMLET for more than 10 cycles (**Figure 6** – green hatched line). This suggests both that HAMLET inhibits increased methicillin-resistance upon methicillin exposure and that *S. aureus* is unable to develop resistance to HAMLET's adjuvant activity.

## Discussion

HAMLET has well documented antibacterial properties against the gram-positive bacterium *Streptococcus pneumoniae*, as well as against strains of *H. influenzae* and some strains of *M. catarrhalis* in vitro, but fails to kill most other bacterial species [23]. HAMLET, used at sublethal concentrations was recently observed to also potentiate the effect of common antibiotics [26]. The purpose of this study was to demonstrate that even though HAMLET showed no antimicrobial activity against any of the *S. aureus* strains used, HAMLET acted as an effective antimicrobial adjuvant with the ability to increase the efficacy of a broad range of commonly used antibiotics including methicillin, vancomycin, erythromycin, and gentamicin, to the degree that drug-resistant *S. aureus* could again become sensitive to these antibiotics both in *in vitro* assays determining MICs and MBCs, as well as for eradication of biofilms and nasopharyngeal colonization *in vivo*. Similarly, we showed that the mechanism of HAMLET-induced potentiation of various antibiotics was similar between *S. pneumoniae* and *S. aureus*. This shows that HAMLET has the ability to act as an adjuvant to potentiate the activity of antibiotics irrespective of its ability to be bactericidal, and suggests that HAMLET may have the potential to act as an adjuvant on a wide range of bacterial species, including species with widespread multi-drug resistance where treatment options are rapidly becoming limited.

Treatment of MRSA infections depends on the clinical situation, the administration route, and the resistance pattern of the organism, but normally entails the use of drugs such as vancomycin, linezolid, daptomycin, clindamycin, and mupirocin [32], [33]. *In vitro* testing has shown some synergy in time-kill assays between daptomycin and oxacillin and a slight increase in eradication of MRSA biofilms with vancomycin in the presence of rifampicin and tigecycline [34]–[36]. Additionally, non-bactericidal inhibitors of efflux pumps have been tested in *S. aureus* for their ability to lower MICs of antibiotics with some success [10], [37]. Although the daptomycin/oxacillin synergy was comparable to the potentiation effect of HAMLET on methicillin, HAMLET has a much stronger potentiation effect in eradicating biofilms, even at relatively low concentrations (6–30 μM, which amounts to 100–500 µg/mL as HAMLET is a protein complex. These concentrations are well within the physiological range found in human milk (2,000 µg/mL; [38], [39])). *In vivo* treatment is primarily based on the use of vancomycin with linezolid and clindamycin as main adjunctive therapies and daptomycin is the antibiotic of choice in cases of vancomycin insensitivity, but both *in vitro* assays and *in vivo* treatment show that antibiotic combination treatment of MRSA invariably result in increased resistance of the agents used, producing increasingly multi-drug resistant strains, which continuously escalate the problems with MRSA treatment [33], [35], [36].

The adjuvant activity of HAMLET presented in this study has three benefits in this regard. First, we showed that we could significantly decrease nasopharyngeal colonization with MRSA with just one administration of methicillin in the presence of HAMLET for 12 hours. A topical decolonization model was chosen both based on the difficulty achieving decolonization clinically to prevent infection [40], as MRSA is a major cause of mucosal infections, and as HAMLET is a non-covalently associated protein-lipid complex that is less effective when administered systemically, where serum proteins compete for binding of the lipid component. Methicillin was used for the studies mainly to verify that HAMLET can reverse the resistance of this agent, that together with penicillins was safely employed topically early in its investigative history [41]–[45]. Unfortunately, since then the rapid emergence of methicillin-resistant staphylococci has almost completely negated this value and practice [32] and other antibiotics such as erythromycin or gentamicin that were once widely used topically have met a similar fate. Thus, our results show that HAMLET can make methicillin and maybe other, currently unusable, antibiotics useful for treatment of mucosal surfaces and have the potential to offer a return to these safer agents for topical use.

Second, part of the mechanism for the increased adaptation of MRSA to ever-increasing levels of oxacillin with parallel increases of resistance to erythromycin, kanamycin, rifampicin and other antibiotics was recently shown to be associated with activation of efflux pumps [31] that are sensitive to agents that dissipate the proton motive force, such as CCCP [37], [46]–[49]. CCCP is known to lower the MIC of fluoroquinolones, tetracyclines and other antibiotics due to its inactivation of efflux pumps that is driven by the proton motive force [37], [50], [51], but CCCP is so toxic that no molecules belonging to this family of energy-inhibitors has been developed for clinical use. HAMLET have a major advantage in this regard, as it dissipates the proton motive force as effectively as CCCP but shows no toxicity to healthy human cells [52]–[55] or to the *S. aureus* cells, indicating that HAMLET's effect is more targeted and useful for drug development. It also has a major advantage as it does not directly interfere with efflux pump function as other potential adjuvants do [10], [37], but causes both a rapid and sustained dissipation of the trans-membrane proton gradient and an influx of calcium through a sodium-dependent mechanism that induces dissipation of the electrical gradient without killing the organism, both of which were required for HAMLET's potentiation effect. This dual membrane effect alters membrane ion gradients required for the activity of efflux pumps, as well as other resistance mechanisms that may explain HAMLET's ability to reverse the resistance of a broad range of antibiotics in a broad range of species besides *S. aureus* [26]. Thus HAMLET may also affect target site alterations, enzymatic inactivation of antibiotic compounds, activity of beta-lactams on penicillin-binding proteins, and indirectly ATP production required for ATP-binding cassette-type multidrug transporters to pump drugs out of the cell, all of which are sensitive to the chemical environment of the cellular membrane [29], [30], [56], [57]. The HAMLET-induced changes of the chemical membrane environment was therefore, not surprisingly, accompanied by an increase in the bacterial association of gentamicin, vancomycin and the beta-lactam Bocillin FL, with resistant strains accumulating more antibiotics. This suggests that HAMLET-induced disruption of the proton motive force and ion gradients also acts by increasing antimicrobial penetration and binding.

The third benefit of HAMLET's dual and potentially parallel actions on the bacterial membrane function is its ability to inhibit MRSA resistance-development after exposure to increasing methicillin concentrations. The presence of HAMLET during methicillin-pressure significantly decreased the resistance development of the MRSA strain used. Additionally and importantly, even after continuous growth in HAMLET, *S. aureus* did not become insensitive to HAMLETs adjuvant properties. Instead the strain that was continually grown in HAMLET plus methicillin over many cycles showed both less resistance-increase to methicillin, and that the strain could still be equally sensitized in the presence of HAMLET. This suggests that HAMLET is not susceptible to the development of resistance and have the potential to reduce the emergence of multi-resistant mutants associated with increased efflux pump function or other resistance mechanisms [58]–[60]. These results agree with findings in *S. pneumoniae* where tests of spontaneous mutation frequency have demonstrated that strains are unable to develop resistance to HAMLET (7).

Recent pilot studies *in vivo* for the local treatment of skin papillomas and bladder cancer using intravesicular instillation of the HAMLET complex have revealed no toxic side effects for *in vivo* use in either animals or humans [52]–[55]. The development of more stable congeners of HAMLET or other HAMLET agonists targeting HAMLET's pathway will likely expand the potential of HAMLET co-therapy to provide a safe and novel approach as an adjuvant to a broader range of currently existing antibiotic therapies. These adjuvant properties of HAMLET are not limited to staphylococci as all bacterial species tested, including gram-positive and gram-negative species *Streptococcus pneumoniae*, *Moraxella catarrhalis*, and *Acinetobacter baumanni*, are similarly sensitive to the potentiating effects of HAMLET [26].

In summary, we present a novel antimicrobial adjuvant that works in combination with existing antimicrobial therapies against multi-drug-resistant staphylococci both *in vitro* and *in vivo*. Further studies are needed to unravel both the molecular mechanisms of antibiotic sensitization induced by HAMLET as well as the development of more stable congeners to exploit its potential as an antibiotic resistance-modifying agent. Its safety, efficacy, specificity, quick onset of action, and low risk for resistance underscore its therapeutic potential that could ultimately reduce levels of antimicrobial resistance in both hospitals and the community, allowing hard-to-treat bacterial infections to be successfully controlled and potentially ‘resuscitating’ old or discarded antibiotics and allow for their deployment for a broader bacterial spectrum.

## Materials and Methods

### Ethics statement

This study was carried out in strict accordance with the recommendations in the Guide for the Care and Use of Laboratory Animals of the National Institutes of Health. The protocol was approved by the Institutional Animal Care and Use Committee at the University at Buffalo, Buffalo, NY, USA (Protocol number: MIC36048Y). All bacterial inoculations and treatments were performed under conditions to minimize any potential suffering of the animals.

### Reagents

Cell culture reagents, Bocillin FL and the AlexaFluor 488 labeling kit were from Invitrogen, Carlsbad, CA. Bacterial and cell culture media, and supplies were from VWR Inc, Radnor, PA. Sheep Blood was purchased from BioLink, Inc, Liverpool, NY. All antibiotics and remaining reagents were purchased from Sigma-Aldrich, St. Louis, MO. Methicillin and gentamicin stocks were suspended in water while erythromycin and vancomycin stocks were dissolved in ethanol. Antibiotic stocks were diluted at least 100-fold in phosphate buffered saline (PBS), pH 7.4, before use in the assays.

### Production of HAMLET

HAMLET was produced by converting EDTA-treated, partially unfolded alpha-lactalbumin in the presence of oleic acid (C18:1) on an anion-exchange matrix to a stable protein-lipid complex, as described [17] and was resuspended in PBS for all experiments.

### Bacterial Strains

Staphylococcal strains were grown in Tryptic Soy Broth (TSB) as described [61]. The use of MRSA and VISA strains of *S. aureus* was approved by the Biosafety Committee at the University at Buffalo, SUNY. Network on Antimicrobial Resistance in *Staphylococcus aureus* (NARSA) strains NRS 1, 70, 71, 100, 123, 384 (all MRSA strains), the MRSA strain 10307570, and the MSSA strain 11090306 were generously provided to us by Dr. Alan Lesse, University at Buffalo, SUNY.

### *In vitro* susceptibility tests

Minimal inhibitory concentrations (MICs) were determined in 96-well microtiter plates using the microdilution method according to approved standards of the CLSI as described previously [62]. However, rather than Mueller-Hinton broth, Tryptic soy broth (TSB) was used for susceptibility testing as it is the common media used for Staphylococcal growth, and as it has shown more consistent results for a variety of antimicrobials when tested against Staphylococci [63]–[65]. This was true also for HAMLET as when traditional Mueller Hinton broth was used, approximately 20% more HAMLET was required for equivalent adjuvant activity as seen in TSB. A two-fold dilution series of antibiotics in triplicate and in the presence or absence of HAMLET was prepared in 96 well microtiter plates, and each well was seeded with a final bacterial concentration of 10^5^ CFU/mL, and was incubated for 24 h at 37°C in a Synergy 2 microplate reader (Biotek, Winooski, VT) where the Optical Density at 600 nm (OD_600_) was recorded every 5 minutes to monitor bacterial growth. The MIC was defined as the lowest concentration of antimicrobial agent solution where no increase in OD_600_ was detected. For MIC assays involving ion inhibitors, Ruthenium Red (RuR; 30 μM) or Amiloride (1 mM), were used along with a concentration of methicillin (4 μg/mL or 10 µM) that only completely inhibited growth in combination with HAMLET (100 μg/mL or 6 µM).

Minimal bactericidal concentrations (MBCs) were determined as described previously [62] from the MIC assay plates, by plating 10 µL of broth from all wells without visible growth as well as from the wells with the highest concentration of antimicrobials that still showed visible growth onto sheep blood agar plates. The MBC was defined as the lowest concentration of antimicrobials yielding colony counts <0.1% (3 log_10_ reduction) of the initial inoculum (as determined by colony counts from the growth control well immediately after inoculation) as described [62].

### Static Biofilm Model

Staphylococci were grown in TSB to mid-logarithmic phase (OD_600_  = 0.5), washed, and resuspended in fresh pre-warmed medium to a density of 2×10^4^ CFU in 500 µl volume, and suspensions were used to seed polystyrene 24-well plates. Bacteria were cultured at 37°C for 12 hours, after which biofilms were washed with PBS to eliminate planktonic bacteria and were exposed to TSB with indicated concentrations of HAMLET and/or antibiotics for 24 hours at 37°C. Biofilms were then washed in PBS, sonicated and detached by scraping the surface in the presence of 100 μL PBS followed by a rinse with 100 μL PBS. Collected cells were vortexed twice for 20 seconds at high speed and the dispersed biofilm cells were first observed by microscopy to ensure proper dispersion and then diluted in a 10-fold dilution series where 100 µl of each dilution was plated on blood agar plates to determine viable CFUs per ml after overnight growth at 37°C. Colony counts from plates carrying 20–200 colonies were used to determine the viable counts and are reported as the total number of colony-forming units per biofilm.

### HAMLET potentiation of Gentamicin and Penicillin in vivo

Six-week-old female BALB/cByJ mice from Jackson Laboratories (Bar Harbor, ME, U.S.A.) were maintained in filter-top cages on standard laboratory chow and water *ad libitum* until use.

Mice were colonized as described previously [66]. In short, 10 µl of a bacterial suspension containing 5x10^7^ CFUs of NRS 70 staphylococci in PBS were pipetted into the nares of non-anesthetized mice. After 24 hours, mice were treated with 20 μl methicillin (0–100 µg or 0–5,000 μg/mL) in the presence or absence of 20 µl (100 μg) HAMLET in the nares for 12 hours. Colonization burden was then assessed after euthanizing the animals by enumerating viable bacteria both in a nasopharyngeal lavage obtained by injecting 100 μL PBS in the trachea of mice and collecting it as it flowed out the nares, and from harvested nasopharyngeal tissue after the nasal wash. Nasopharyngeal tissue was dissected out as described [26], [67] by removing the upper skull bone, and harvesting the tissue present in the nasal conchae with forceps. Bacterial load was measured by determining viable plate counts from the nasal lavage or from homogenized tissue as described above.

### Assessing membrane potential

Staphylococci grown to late log phase in TSB were pelleted by centrifugation at 2,400×*g* for 10 minutes and washed twice by resuspension in PBS. The bacterial pellet was resuspended in PBS to half of the original volume and energized with 50 mM glucose for 15 minutes at 37°C. To measure membrane potential of the staphylococcal membrane, 500 nM DiBAC_4_(3) (bis-(1,3-dibutylbarbituric acid) trimethine oxonol; Molecular Probes, Eugene, OR, USA) were added. In a 96-well plate, a 100 μL volume of this bacterial suspension was then added to 100 μL of PBS containing vehicle alone or HAMLET and antibiotic combinations in the presence or absence of specific ion transport inhibitory compounds yielding a final concentration of 25 mM glucose and 250 nM DiBAC_4_(3) per well. Triton X −100 (0.1%; Sigma-Aldrich) was used as a positive control for membrane depolarization and rupture. The plate was then placed immediately into a pre-warmed (37°C) Synergy 2 Multi-Mode Microplate Reader (BioTek) where fluorescence readings from DiBAC_4_(3) (485/20 nm excitation, 528/20 nm emission) were taken every minute for one hour. The difference in fluorescence intensity between the untreated control and the HAMLET-treated sample was calculated for the “no inhibitor” samples and for the “inhibitor” samples using the values after 60 minutes. The fluorescence intensity difference for the “inhibitor” samples was then expressed as fold change of the intensity compared with the HAMLET alone (“no inhibitor”) sample, providing the degree of depolarization compared to the “HAMLET alone” sample.

### Radioisotope ^45^Ca^2+^ transport assays

*Staphylococcus aureus* was grown to log phase, washed three times and resuspended in 1XPBS containing 0.5 mM CaCl_2_ (CaPBS). No glucose was added in order to minimize the interference created by extrusion of Ca^2+^ via ATPase pumps. ^45^CaCl_2_ (PerkinElmer; Waltham, MA, USA) was added to the cells at a final concentration of 2.5 µCi/mL, followed by inhibitor compound, with each addition given two minutes equilibration time. The untreated baseline sample was measured at this point. The sample was then divided, HAMLET was added to one of the tubes, and ^45^Ca^2+^ uptake was measured at various intervals. For each sample, 100 µL was dispensed onto Millipore 0.3 µm PHWP filters (EMD Millipore; Billerica, MA, USA) presoaked in CaPBS, and immediately washed with 9 mL of CaPBS via syringe filtration through Millipore Swinnex**®** filter holders. Filters were placed in scintillation vials with 5 mL of scintillation fluid, and CPMs were detected on a Wallac 1409 liquid scintillation counter (Wallac Oy, Turku, Finland). The results were expressed as ΔCPM that was calculated for each sample at the indicated time points as the difference between HAMLET-treated and untreated samples.

### Intracellular pH measurement

A previously reported protocol for intracellular pH (pH_i_) measurement [68] was modified and optimized for *S. aureus* by testing the effect of various dye concentrations and staining times on the measured fluorescence of *S. aureus* isolate NRS 384 grown to mid-log phase. Cell samples were subjected to increasing concentrations of the membrane-permeant acetoxymehtyl (AM) ester derivative of the dual-excitation ratiometric pH indicator dye BCECF (2′,7′-bis-(2-carboxyethyl)-5-(and-6)- carboxyfluorescein), for 30 minutes at 30°C. Fluorescence was measured as a ratio of fluorescence at 530 nm with dual wavelength excitation at 490 and 440 nm and calibration curves were established for each independent experiment. After testing a number of loading concentrations we observed that the fluorescence ratios remains generally constant when concentrations above 20 µM was used. Therefore 25 μM BCECF-AM was used for all experiments. The optimized staining time, 30 minutes, was determined using the same criteria and was used for all experiments. After loading, the cells were washed twice in PBS by centrifugation and the resulting pellet was resuspended in PBS to the original volume and HAMLET and HAMLET/antibiotic combinations were added. CCCP was added as a positive control. CCCP is a lipophilic weak acid that is soluble in the lipid domain of the membrane in both the protonated and deprotonated form. This allows it to act as a protonophore causing an influx of H^+^ into the cytoplasm, dissipating both the electrical potential and the H^+^ gradient across the inner membrane. For each experiment calibration was performed where cells were resuspended in high [K^+^] buffers at different pH values ranging from 6.5 to 8.0 [69]. Nigericin (20 µM; a potassium/hydrogen antiport with some ionophore activity for both ions) and valinomycin (20 µM; a potassium ionophore) were added in combination to the samples to equilibrate the pH_i_ of the cells to the pH of the surrounding buffer as described [69] both for the creation of a standard curve during calibration, and at the end of each experiment to demonstrate the pH of the experimental buffer.

### Membrane Integrity Assays

To evaluate the effect of HAMLET on membrane integrity, Staphylococci were exposed to HAMLET and antibacterial agents at indicated concentrations for 50 minutes at 37°C in PBS. The detergent Triton X-100 (0.1%) was used as a control, known to cause extensive membrane rupture. After treatment the leakage of soluble components (DNA RNA and protein) were measured from supernatant obtained after centrifugation of the bacterial suspension at 13,000×g for 5 min, by measuring the absorbance at 260 nm in a BioTek Synergy 2 plate reader with the Take3 microdrop addition (Biotek). Cellular release of the small molecule ATP (MW 507) that was measured from the same supernatants using the ATP determining kit (Invitrogen), which measures the luminescence of oxyluciferin produced from the oxidation of luciferin by luficerase, a process that requires ATP degradation. The uptake of the membrane impermeable DNA binding dye propidium iodide (MW 668) was measured by the fluorescence intensity of the whole bacterial suspension after 50 min in a Synergy 2 Microplate Reader (BioTek; 528/20 nm excitation, 605/20 nm emission). Where applicable, independent readings were also taken in the presence of antibacterial agents alone to enable corrections for potential background leakage.

### Conjugation of Gentamicin with Alexa Fluor 488

Conjugation of gentamicin with AlexaFluor 488 was performed using the AlexaFluor 488-conjugation kit (Invitrogen), adapted from the manufacturer's instructions as described [26]. In short, AlexaFluor 488 ester was added to a rapidly stirred solution of 0.1 M sodium bicarbonate, pH 8.3 and 10 mg/mL gentamicin, and was incubated for 5 hours at 4°C. A gentamicin/AlexaFluor molar ratio of 10:1 was used to minimize formation of multiply substituted Alexa-Fluor 488-gentamicin conjugates. Conjugated gentamicin was separated from unreacted dye using a provided desalting resin. Conjugation efficiency was determined by measuring the moles of dye per mole gentamicin. The absorbance of the conjugated antibiotic was determined at 494 nm and divided by 71,000 M^−1^cm^−1^, the molar extinction coefficient for AlexaFluor 488 at 494 nm, and the concentration of gentamicin in the sample (estimated based on a minimum retention of 80% after purification over the resin). The conjugation efficiency was 1.2 moles AlexaFluor 488 per mole gentamicin in the batch used. The conjugated gentamicin was shown to retain its anti-microbial activity and was stored at 4°C until used.

### Gentamicin, Vancomycin and Bocillin FL Binding

We adapted a previously described method [70] using AlexaFluor 488-gentamicin, vancomycin FL and Bocillin FL as reporter antibiotics to investigate association of these compounds with the staphylococcal cells. In brief, indicated strains were grown in TSB to an OD_600nm_ of 0.5. Antibiotics were added at the following concentrations: Gentamicin at 50 μg/mL, Vancomycin FL at 20 µg/mL, Bocillin FL at 25 μg/mL alone, or in combination with HAMLET at 100 µg/mL.

After 30 minutes, cultures were centrifuged at 9,000×*g* for 4 min, washed four times with PBS, and resuspended in a small volume of PBS. Cells were lysed by 5 successive freeze thaw cycles at −80°C and 37°C for 5 min each as described previously [57]. The Bocillin FL, vancomycin FL or Alexa Fluor 488-gentamicin concentration was determined by measuring the amount of fluorescent material in a Synergy 2 microplate reader (Biotek) using excitation and emission wavelengths set at 485 and 530 nm, respectively. Linearity was obtained between 2 and 800 μg/mL; (R^2^  = 0.9983) for AlexaFluor 488-gentamicin, between 0.5 and 100 μg/mL; (R^2^  = 0.9928) for Bocillin FL, and between 0.25 and 50 µg/mL; (R^2^  = 0.9919) for Vancomycin FL, with binding expressed as a ratio of the fluorescence of each sample divided with the sample protein content, determined by the 280/260 nm ratio using the using a BioTek Synergy 2 plate reader with the Take3 microdrop addition (Biotek).

### HAMLET and Methicillin Resistance Development

Experiments were designed to test the impact of HAMLET on the adaptation of MRSA to methicillin in liquid cultures, and to test HAMLET's potential to inhibit resistance increase during antibiotic pressure. Series of tubes containing two-fold increasing concentrations of methicillin (1–512 µg/ml or 2.5–1,350 µM) in the absence or presence of HAMLET (100 µg/ml or 6 µM) were incubated with MRSA (NRS 384; 10^7^–10^8^ cfu/ml innocula), as described for MIC determination above. After 12 hours of incubation, 0.1 mL samples from the tubes containing the highest antibiotic concentration that still showed turbidity were used to inoculate a new series of tubes containing the same antibiotic serial dilution series in the presence and absence of HAMLET. The experiments were performed over 10 cycles. After each cycle and additional 0.1 mL bacteria from the tubes with the highest antibiotic concentration that still showed turbidity were washed twice in PBS to eliminate the antibiotics and HAMLET. These bacteria were then used to determine their MIC in the absence or presence of 6 µM HAMLET in a separate assay, as described above. These assays provided the MIC values for Figure****6, and helped us evaluate the effect of HAMLET on adaptation to methicillin and the response of these bacteria to HAMLET's adjuvant activity

### Statistical Analysis

The data were analyzed for statistical significance by a two-tailed Student's t-test for unpaired data. A *P-*value <0.05, was considered significant.
